# Molecular record for the first authentication of *Isaria cicadae* from Vietnam

**DOI:** 10.1515/biol-2021-0074

**Published:** 2021-07-15

**Authors:** Thuan Duc Lao, Hanh Van Trinh, Loi Vuong, Luyen Tien Vu, Thuy Ai Huyen Le, Hiep Minh Dinh, Nguyen Binh Truong

**Affiliations:** Department of Pharmaceutical and Medical Biotechnology, Faculty of Biotechnology, Ho Chi Minh City Open University, Ho Chi Minh City, Vietnam; University of Science, VNU-HCM, Ho Chi Minh City, Vietnam; Institute of Applied Technology, Thu Dau Mot University, Binh Duong, Vietnam; Department of Agriculture and Rural Development of Ho Chi Minh City, Ho Chi Minh City, Vietnam; Faculty of Biology, Dalat University, Lam Dong, Vietnam

**Keywords:** nuclear small ribosomal subunit, nuclear large ribosomal subunit, *Isaria cicadae*, phylogeny

## Abstract

The entomopathogenic fungus T011, parasitizing on nymph of Cicada, collected in the coffee garden in Dak Lak Province, Vietnam, was preliminarily morphologically identified as *Isaria cicadae*, belonged to order Hypocreales and family Clavicipitaceae. To ensure the authenticity of T011, phylogenetic analysis of the concatenated set of multiple genes including *ITS*, *nrLSU*, *nrSSU*, *Rpb1*, and *Tef1* was applied to support the identification. Genomic DNA was isolated from dried sample T011. The PCR assay sequencing was applied to amplify *ITS*, *nrLSU*, *nrSSU*, *Rpb1*, and *Tef1* gene. For phylogenetic analysis, the concatenated data of both target gens were constructed with MEGAX with a 1,000 replicate bootstrap based on the neighbor-joining, maximum likelihood, maximum parsimony method. As the result, the concatenated data containing 62 sequences belonged to order Hypocreales, families Clavicipitaceae, and 2 outgroup sequences belonged to order Hypocreales, genus *Verticillium*. The phylogenetic analysis results indicated that T011 was accepted at subclade *Cordyceps* and significantly formed the monophyletic group with referent *Cordyceps cicadae* (Telemorph of *Isaria cicadae*) with high bootstrap value. The phylogenetically analyzed result was strongly supported by our morphological analysis described as the *Isaria cicadae*. In summary, phylogenetic analyses based on the concatenated dataset were successfully applied to strengthen the identification of T011 as *Isaria cicadae*.

## 1 Introduction

*Isaria cicadae* Miq., Bull. Sci. phys. nat. néerl.: 86 (1838) (Mycobank: MB#204858), also known *Cordyceps cicadae* (Miq.) Massee (1895) (Mycobank: MB#311793), is the entomopathogenic fungi capable of parasitizing on cicada nymph, belongs to the order Hypocreales, and the family Clavicipitaceae [4,5]. *C. cicadae* usually distribute in many regions of the world with temperatures ranging from 18 to 24°C, relative humidity of >80°C, and grows vertically on the sunny slopes at an attitude of 700–950 m [3]. The distribution of *C. cicadae* is recorded in China (Province of Yunan, Sichuan, Guizhou, Jiangsu, Guangdong, Hunan, Hubei, etc.), Korea (Jeju Island), and Japan (South of Fukushima). Furthermore, *C. cicadae* is also seen in Thailand, North America, and Europe [3,4,5,6,7,8,9,10,11,12,13,14,15,16,17,18,19,20,21,22,23,24].

Due to their numerous bioactivities, *I. cicadae*, as well as *C. cicadae*, is considered the most valued traditional Chinese medicine. Its medicinal bioactive components, such as adenosine, cordycepin, ergosterol, etc., which have been used to relieve exhaustion remedy, treat numerous diseases, such as antitumor activities, and food source, have been recorded [3,19,20,21,22]. To obtain precious valued herbal medicine, the exploration and collection of local *I. cicadae* (*C. cicadae*) play an important role to apply for further medicinal applications. During our expedition to validate the fungal diversity in Ea Knop Town – Ea Kar District (Latitude: 12°34′26″N–13°02′09″N; Longitude: 108°22′08″E–108°43′2″E) located in Dak Lak Province, we collected the sample T011, parasitizing on the nymph of Cicada, which was classified and confirmed by the specialist on the entomologist, Faculty of Biotechnology, Ho Chi Minh City Open University, Ho Chi Minh City, Vietnam. In this paper, to ensure the origin and authenticity of T011 as *I. cicadae*, we conducted the morphology analysis and molecular phylogenetic analysis of the concatenated set genes including *ITS*, *nrLSU*, *nrSSU, Rpb1,* and *Tef1*.

## 2 Materials and methods

### 2.1 Sample collection

The specimen T011, parasitizing on the nymph of Cicada, was collected in the coffee garden in Ea Knop Town – Ea Kar District, Dak Lak Province on the morning of June 24, 2018. In the laboratory, the specimen was conditioned to be dried at 60°C and stored for further analysis.

### 2.2 Morphology analysis

Macroscopic characteristics of the fresh body were carefully observed in the many macroscopic characteristics. For the microscopic analysis, a bunch of conidiogenous cells was cut into small species, then, soaked in the water for about 3 min. A sample of the synemata containing the conidiogenous cells was immersed in distilled water for 3 min. Asexual spores were removed using a clean brush. The fertile part was then analyzed under a microscope. Conidia size was recorded. According to the identification of conidia, phialides, and colony coloration, the isolate cultures were grown on YMG media, composed of 4 g/L Yeast extract, 10 g/L Malt extract, 4 g/L Glucose, incubated at 20°C within a period of 20 days.

### 2.3 DNA extraction, PCR amplification, target gene sequencing

Genomic DNA was extracted from dried material by using the phenol/chloroform method (pH = 8). The dried material was added to a lysis buffer (2.0% SDS, Tris-HCl pH 8.0, 150 mM NaCl, 10 mM EDTA, 0.1 mg/mL Proteinase K). During the incubation at 65°C for overnight, it was mixed thoroughly by inverting the tube several times. Then, the supernatant was collected by centrifugation. About 700 μL of phenol/chloroform/isoamyl alcohol at a ratio of 25:24:1 was added and centrifuged. The upper solution was collected, precipitated with absolute ethanol, and washed with 70% ethanol. DNA concentration was identified by using OD_260_. Finally, isolated genomic DNA was stored in Tris-EDTA buffer at −20°C for further studies.

The primer pairs used to amplify *ITS*, *nrLSU*, *nrSSU, Rpb1,* and *Tef1* region were shown in Table 1. The final volume for PCR was done in the total of 15 μL with the thermal program: 1 cycle for 95°C for 5 min; 40 cycles of 95°C for 30 s, *X*°C for 30 s, 72°C for 2 min; 1 cycle for 72°C for 5 min (Note: *X*°C is the annealing temperatures for each target gene, shown in Table 1). About 5 μL aliquots of amplification products were electrophoresed on a 2.0% agarose gel and visualized in a UV transilluminator. The amplified product was sequenced at Nam Khoa (Vietnam) company.

**Table 1:** The primers’ sequences used in this study

Target gene	Primer	Sequence (5′–3′)	*T*_a_ (°C)	Reference
*nrLSU*	LR0R (F)	GTACCCGCTGAACTTAAGC	55	[18]
	LR5 (R)	ATCCTGAGGGAAACTTC		
*nrSSU*	NS1 (F)	GTAGTCATATGCTTGTCTC	42.2	[20]
	NS4 (R)	CTTCCGTCAATTCCTTTAAG		
*ITS*	ITS1F	CTTGGTCATTTAGAGGAAGTAA	55	[20]
	ITS4	TCCTCCGCTTATTGATATGC		
*Rpb1*	CRPB1	CCWGGYTTYATCAAGAARGT	46.3	[13]
	RPB1Cr	CCNGCDATNTCRTTRTCCATRTA		
*Tef1*	983F	GCYCCYGGHCAYCGTGAYTTYAT	55	[2]
	2218R	ATGACACCRACRGCRACRGTYTG		

Note: F: forward primer; R: reverse primer; *T*_a_: annealing temperature.

### 2.4 Taxa and *ITS, nrLSU, nrSSU, Rpb1,* and *Tef1* sequences collection, DNA proofreading, and phylogeny analysis

The data set of *ITS*, *nrLSU*, *nrSSU, Rpb1,* and *Tef1* sequences were established by sequences downloaded from Genbank (NCBI) and based on the previous data published by Sung et al. (2007) [16]. The *ITS*, *nrLSU*, *nrSSU, Rpb1,* and *Tef1* were noted with accession number, name of taxon, and locality. The multiple gene data used in the current study were established based on the combination of *ITS*, *nrLSU*, *nrSSU, Rpb1,* and *Tef1* data. The amplified DNA sequences were proofread to remove ambiguous signals at both ends by different software, including Seaview 4.2.12 and Chromas Lite 2.1.1. The phylogenetic tree was constructed based on the neighbor-joining (NJ), maximum parsimony (MP), and maximum likelihood (ML) by using Molecular Evolutionary Genetics Analysis (MEGA) version X. Additionally, the best evolution model was predicted by using jModelTest.

## 3 Results

### 3.1 Morphology analysis

**Figure 1:**
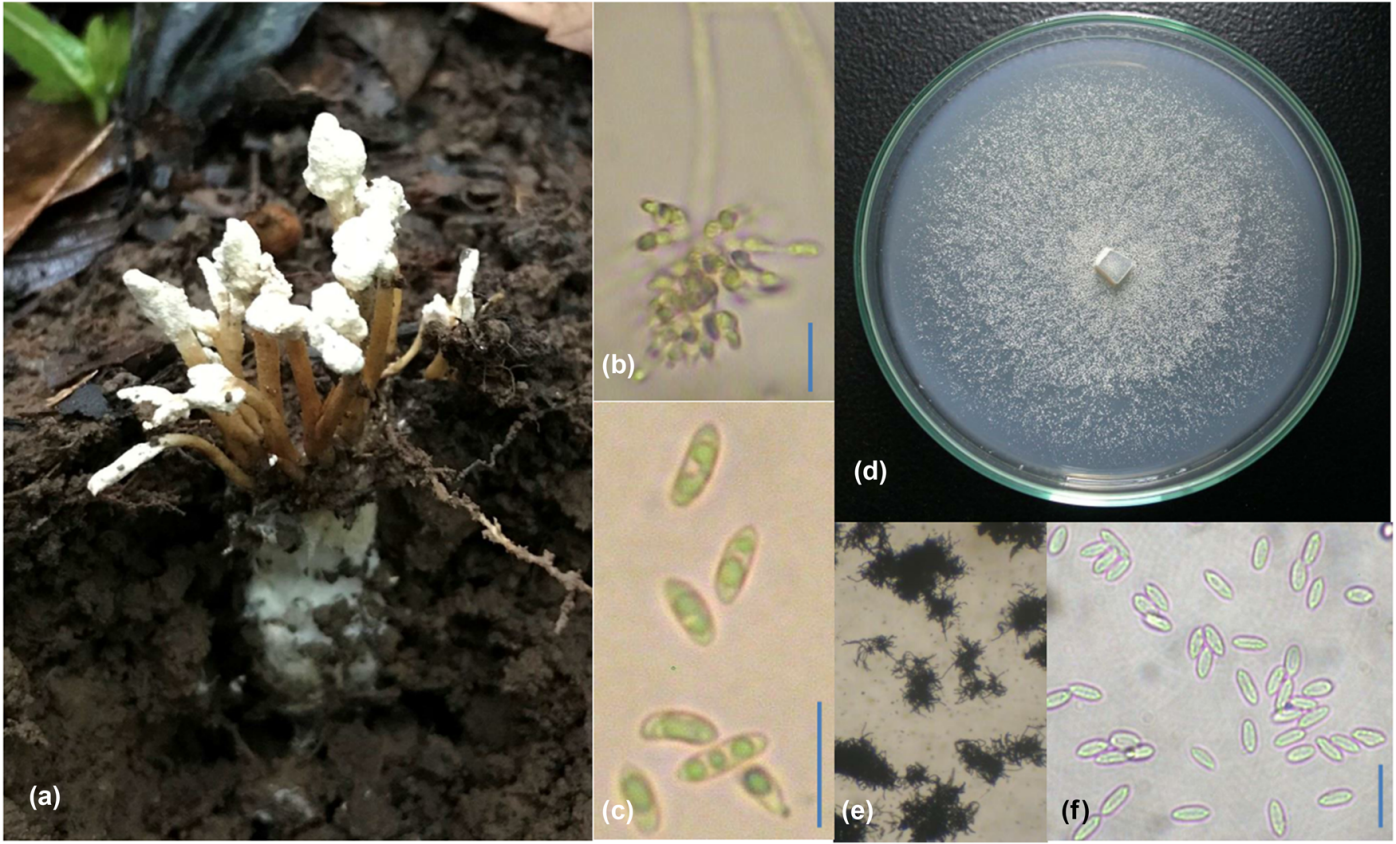
Morphology of *Isaria cicadae*: (a) Synemata forming from Cicadae, (b) Phialides, (c) conidia, (d) mycelia after 30 days on PDA media, (e) chain of conidia, and (f) conidia from mycelia. The bar scale indicated 10 µm.

The sample of T011 was collected in the soil of the coffee garden on the morning of June 24, 2018. The synnemata were emerging from the soil, while the host was in the soil. **Host:** unidentified cicada nymph. **Synnemata:** presence, branching, 15–60 mm in length × 1.0–2.5 mm in diameter. Synnemata originated from the head of cicada nymphs with the thick layer of mycelia (hiding under the ground). **Color:** white to cream. **Form:** simple, erect, and distinguishing form fertile part and stipe. **Fertile part:** a dense white powdery covering on the surface due to the presence of a mass of conidia. **Phialides:** grouped inside the fertile part. Conidia: hyaline to white, cylindrical, 4.7–6.5 µm in length × 2.6–3.1 µm in diameter. **Colonies from cultures on PDA:** floccose and white, then becoming powdery by the conidiation of aerial hyphae. **Conidia from the mycelia:** smaller than those of from synnemata (Figure 1).

### 3.2 Amplification of *ITS, nrLSU, nrSSU, Rpb1,* and *Tef1* gene

Isolated genomic DNA was amplified with the described primers; then, electrophoresis on 2.0% agarose gel showed a significant and clear band of gene *ITS*: 700 bps*, nrLSU*: 950 bps, *nrSSU*: 1,102 bps, *Rpb1*: 803 bps, and *Tef1*: 1,020 bps. The PCR product was sequenced. Sequencing signals of both strands of both target genes were unique and good for reading (data not shown). According to BLAST results, the *ITS, nrLSU, nrSSU, Rpb1,* and *Tef1* of T011 were similar to *ITS, nrLSU, nrSSU, Rpb1,* and *Tef1* of *C. cicadae* (Telemorph of *I. cicadae*) (Table 2).

**Table 2:** BLAST results of T011 specimen’s *ITS, nrLSU, nrSSU, Rpb1,* and *Tef1*

Target gene	BLAST description	Total score	Per. Ident.	*E*-value	Accession
*nrLSU-F*	*Cordyceps cicadae*	1,509	100.00	0.0	MH879588
*nrLSU-R*	*Cordyceps cicadae*	1,509	99.64	0.0	MH879588
*nrSSU-F*	*Cordyceps cicadae*	1,109	100.00	0.0	MH879636
*nrSSU-R*	*Cordyceps cicadae*	1,048	99.65	0.0	MH879636
*ITS-F*	*Cordyceps cicadae*	1,000	99.82	0.0	MT555324
*ITS-R*	*Cordyceps cicadae*	1,444	99.82	0.0	MN128643
*Tef1-F*	*Cordyceps cicadae*	1,729	99.17	0.0	MH879662
*Tef1-R*	*Cordyceps cicadae*	1,676	98.33	0.0	MN576985
*Rpb1-F*	*Cordyceps cicadae*	1,247	100.00	0.0	MN913552
*Rpb1-F*	*Cordyceps cicadae*	1,280	99.57	0.0	MN576876

Note: F: forward sequence; R: reverse sequence; Per. Ident.: percentage of identity.

### 3.3 The systematic concatenated *ITS, nrLSU, nrSSU, Rpb1,* and *Tef1* dataset and phylogeny analysis

**Figure 2:**
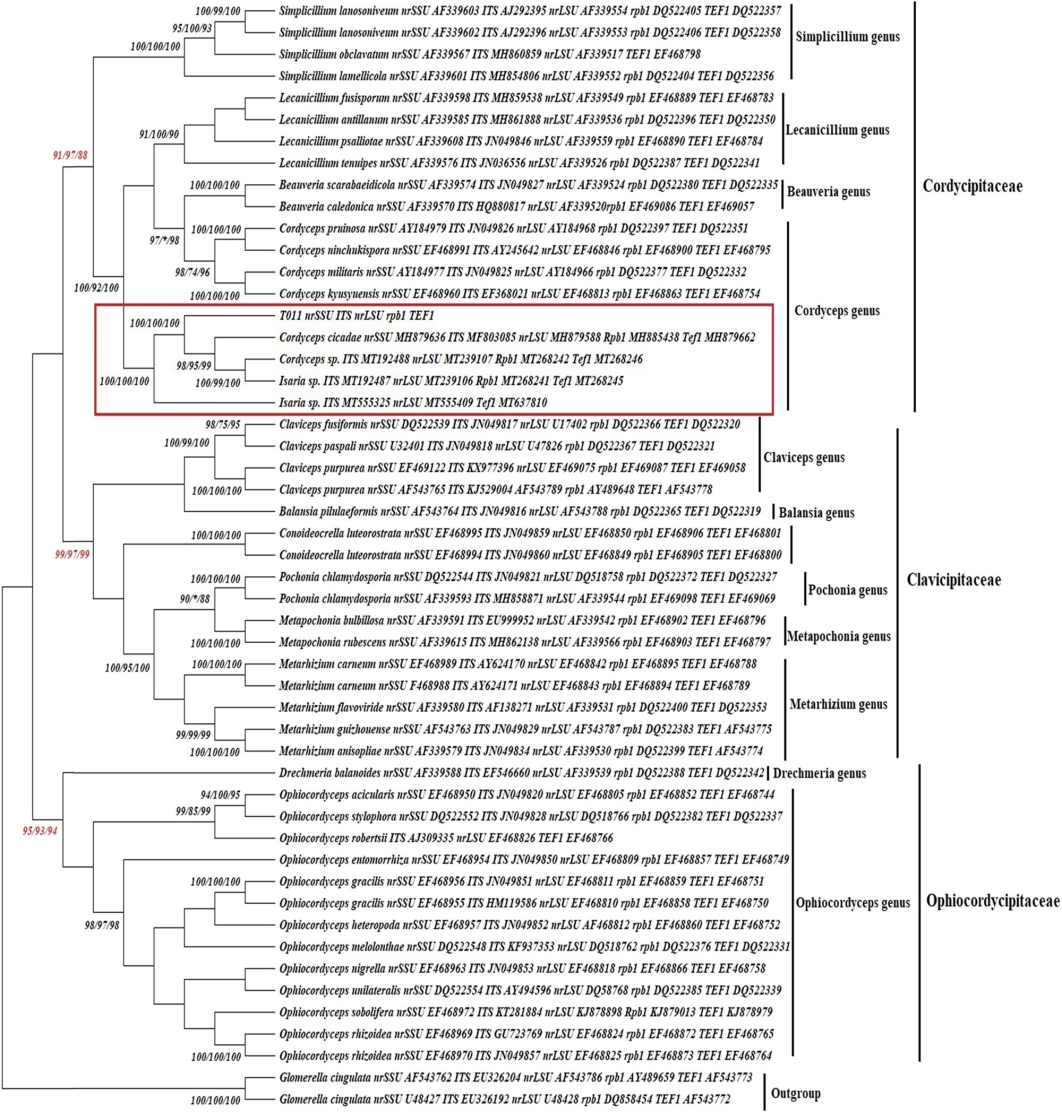
Phylogenetic relationships among T011 and concatenated data set based on the analysis of ML method with bootstrap 1,000. The support values associated with each internal branch correspond to NJ, MP, ML method, indicating that T011 was closely related to *C. cicadae* (Teleomorph of *I. cicadae*).

Total of 62 sequences of *ITS, nrLSU, nrSSU, Rpb1,* and *Tef1* belonged to order Hypocreales, families Clavicipitaceae (served as referent data), and 2 sequences belonged to order Hypocreales, genus *Verticillium* (served as outgroup) were collected from Genbank and listed in Table 3 and T011 sequence. According to 62 sequences, they were divided into three families (Cordycipitaceae, Clavicipitaceae, Ophiocordycipitaceae), and each family was also divided intro genus, including Cordycipitaceae (genus: *Cordyceps, Beauveria, Simplicillium, Lecanicillium*), Clavicipitaceae (genus: *Claviceps, Balansia, Pochonia, Conoideocrella, Metapochonia, Metarhizium*), and Ophiocordycipitaceae (genus: *Drechmeria, Ophiocordyceps*). The best-fit model of DNA evolution for the analyses was obtained using the jModelTest2. Results are shown from General Time Reversible and Gramma distributed with invariant Sites (G + I) with the following parameters: parameters = 109, BIC = 42628.811, lnL = −20671.999, (+I) = 0.450, (+G) = 0.466, R = 2.186, f(A) = 0.246, f(T) = 0.221, f(G) = 0.257, f(C) = 0.276, r(AT) = 0.030, r(AC) = 0.040, r(AG) = 0.110, r(TA) = 0.030, r(TC) = 0.260, r(TG) = 0.040, r(CA) = 0.040, r(CT) = 0.220, r(CG) = 0.050, r(GA) = 0.100, r(GT) = 0.330, and r(GC) = 0.040. This model was used to construct phylogenetic trees using maximum likelihood from concatenated data set. Phylogenetic analysis was presented in Figure 2. As the results, the Clavicipitaceae formed a strong monophyletic group and separated from the out group. All the species in our dataset formed threes clades that were previously reported. According to T011, the T011 multiple gene sequences (*ITS, nrLSU, nrSSU, Rpb1,* and *Tef1*) formed a group with referent sequences of *C. cicadae, Cordyceps* sp., and *Isaria* sp., belonged to the clade Clavicipitaceae, subclade Cordyceps, with the high supported bootstrap values: 100, 100, 100 for NJ, MP, ML method (Figure 2, blanket). Therefore, the molecular identification indicated that T011 was identified as *I. cicadae* (anamorph *of C. cicadae*).

**Table 3:** The concatenated dataset of *ITS, nrLSU, nrSSU, Rpb1,* and *Tef1* genes used for the construction of phylogenetic trees

No.	Taxon	Genus	Accession				
			*nrLSU*	*nrSSU*	*Rbp1*	*Tef1*	*ITS*
1	*Balansia pilulaeformis*	*Balansia*	AF543788	AF543764	DQ522365	DQ522319	JN049816
2	*Beauveria caledonica*	*Beauveria*	AF339520	AF339570	EF469086	EF469057	HQ880817
3	*Beauveria scarabaeidicola*	*Beauveria*	AF339524	AF339574	DQ522380	DQ522335	JN049827
4	*Beauveria staphylinidicola*	*Beauveria*	EF468836	EF468981	EF468881	EF468776	—
5	*Claviceps fusiformis*	*Claviceps*	U17402	DQ522539	DQ522366	DQ522320	JN049817
6	*Claviceps paspali*	*Claviceps*	U47826	U32401	DQ522367	DQ522321	JN049818
7	*Claviceps purpurea*	*Claviceps*	AF543789	AF543765	AY489648	AF543778	KJ529004
8	*Claviceps purpurea*	*Claviceps*	EF469075	EF469122	EF469087	EF469058	KX977396
9	*Conoideocrella luteorostrata*	*Conoideocrella*	EF468850	EF468995	EF468906	EF468801	JN049859
10	*Conoideocrella luteorostrata*	*Conoideocrella*	EF468849	EF468994	EF468905	EF468800	JN049860
11	*Cordyceps cardinalis*	*Cordyceps* sp.	AY184963	AY184974	EF469088	EF469059	—
12	*Cordyceps* cf*. pruinosa*	*Cordyceps*	EF468820	EF468965	EF468868	EF468760	—
13	*Cordyceps* cf*. pruinosa*	*Cordyceps*	EF468821	EF468966	EF468869	EF468762	—
14	*Cordyceps* cf*. pruinosa*	*Cordyceps*	EF468823	EF468968	EF468871	EF468761	—
15	*Cordyceps cicadae*	*Cordyceps*	MH879588	MH879636	MH885438	MH879662	MF803085
16	*Cordyceps kyusyuensis*	*Cordyceps*	EF468813	EF468960	EF468863	EF468754	EF368021
17	*Cordyceps militaris*	*Cordyceps*	AY184966	AY184977	DQ522377	DQ522332	JN049825
18	*Cordyceps pruinosa*	*Cordyceps*	AY184968	AY184979	DQ522397	DQ522351	JN049826
19	*Cordyceps* sp.		MT239107	—	MT268242	MT268246	MT192488
20	*Drechmeria balanoides*	*Drechmeria*	AF339539	AF339588	DQ522388	DQ522342	EF546660
21	*Drechmeria zeospora*	*Drechmeria*	AF339589	—	EF469091	EF469062	—
22	*Isaria* sp.		MT239106	—	MT268241	MT268245	MT192487
23	*Isaria* sp.		MT555409	—	—	MT637810	MT555325
24	*Lecanicillium antillanum*	*Lecanicillium*	AF339536	AF339585	DQ522396	DQ522350	MH861888
25	*Lecanicillium fusisporum*	*Lecanicillium*	AF339549	AF339598	EF468889	EF468783	MH859538
26	*Lecanicillium psalliotae*	*Lecanicillium*	AF339559	AF339608	EF468890	EF468784	N049846
27	*Lecanicillium tenuipes*	*Lecanicillium*	AF339526	AF339576	DQ522387	DQ522341	JN036556
28	*Metarhizium guizhouense*	*Metarhizium*	AF543787	AF543763	DQ522383	AF543775	JN049829
29	*Pochonia chlamydosporia*	*Pochonia*	DQ518758	DQ522544	DQ522372	DQ522327	JN049821
30	*Metapochonia bulbillosa*	*Metapochonia*	AF339542	AF339591	EF468902	EF468796	EU999952
31	*Metapochonia rubescens*	*Metapochonia*	AF339566	AF339615	EF468903	EF468797	MH862138
32	*Metarhizium anisopliae*	*Metarhizium*	AF339530	AF339579	DQ522399	AF543774	JN049834
33	*Metarhizium carneum*	*Metarhizium*	EF468842	EF468989	EF468895	EF468788	AY624170
34	*Metarhizium carneum*	*Metarhizium*	EF468843	EF468988	EF468894	EF468789	AY624171
35	*Metarhizium flavoviride*	*Metarhizium*	AF339531	AF339580	DQ522400	DQ522353	AF138271
36	*Ophiocordyceps acicularis*	*Ophiocordyceps*	EF468805	EF468950	EF468852	EF468744	JN049820
37	*Ophiocordyceps acicularis*	*Ophiocordyceps*	EF468804	EF468951	EF468853	EF468745	
38	*Ophiocordyceps aphodii*	*Ophiocordyceps*	DQ518755	DQ522541	—	DQ522323	—
39	*Ophiocordyceps entomorrhiza*	*Ophiocordyceps*	EF468809	EF468954	EF468857	EF468749	JN049850
40	*Ophiocordyceps melolonthae*	*Ophiocordyceps*	DQ518762	DQ522548	DQ522376	DQ522331	KF937353
41	*Ophiocordyceps stylophora*	*Ophiocordyceps*	EF468837	EF468982	EF468882	EF468777	—
42	*Ophiocordyceps stylophora*	*Ophiocordyceps*	DQ518766	DQ522552	DQ522382	DQ522337	JN049828
43	*Ophiocordyceps unilateralis*	*Ophiocordyceps*	DQ518768	DQ522554	DQ522385	DQ522339	AY494596
44	*Ophiocordyceps variabilis*	*Ophiocordyceps*	EF468839	EF468985	EF468885	EF468779	—
45	*Ophiocordyceps variabilis*	*Ophiocordyceps*	DQ518769	DQ522555	DQ522386	—	—
46	*Ophiocordyceps gracilis*	*Ophiocordyceps*	EF468810	EF468955	EF468858	EF468750	HM119586
47	*Ophiocordyceps gracilis*	*Ophiocordyceps*	EF468811	EF468956	EF468859	EF468751	JN049851
48	*Ophiocordyceps heteropoda*	*Ophiocordyceps*	AY489722	AY489690	AY489651	AY489617	—
49	*Ophiocordyceps heteropoda*	*Ophiocordyceps*	EF468812	EF468957	EF468860	EF468752	JN049852
50	*Ophiocordyceps nigrella*	*Ophiocordyceps*	EF468818	EF468963	EF468866	EF468758	JN049853
51	*Ophiocordyceps rhizoidea*	*Ophiocordyceps*	EF468825	EF468970	EF468873	EF467764	JN049857
52	*Ophiocordyceps rhizoidea*	*Ophiocordyceps*	EF468824	EF468969	EF468872	EF468765	GU723769
53	*Ophiocordyceps robertsii*	*Ophiocordyceps*	EF468826	—	—	EF468766	AJ309335
54	*Ophiocordyceps sobolifera*	*Ophiocordyceps*	KJ878898	KJ878933	KJ879013	KJ878979	KT281884
55	*Cordyceps ninchukispora*	*Cordyceps*	EF468846	EF468991	EF468900	EF468795	AY245642
56	*Pochonia chlamydosporia*	*Pochonia*	AF339544	AF339593	EF469098	EF469069	MH858871
57	*Simplicillium lamellicola*	*Simplicillium*	AF339552	AF339601	DQ522404	DQ522356	MH854806
58	*Simplicillium lanosoniveum*	*Simplicillium*	AF339554	AF339603	DQ522405	DQ522357	AJ292395
59	*Simplicillium lanosoniveum*	*Simplicillium*	AF339553	AF339602	DQ522406	DQ522358	AJ292396
60	*Simplicillium obclavatum*	*Simplicillium*	AF339517	AF339567	—	EF468798	MH860859
61	*Tolypocladium longisegmentum*	*Tolypocladium*	EF468816	—	EF468864	—	—
62	*Tolypocladium fractum*	*Tolypocladium*	DQ518759	DQ522545	DQ522373	DQ522328	—
63	*Glomerella cingulata*	*Polycephalomyces*	AF543786	AF543762	AY489659	AF543773	EU326204
64	*Glomerella cingulata*	*Polycephalomyces*	U48428	U48427	DQ858454	AF543772	EU326192

## 4 Discussion

Morphology analysis indicated that T011 is *Isaria cicadae*, belonged to the family of Cordycipitaceae. Based on the morphology analysis, our specimen T011 shared the common features of *Isaria cicadae* Miq. Bull. Sci. phys. nat. Néerl.: 85 (1838) [17], including: (1) specimen grew in the soil, (2) parasite on the nymph of cicada, (3) synnemata were simple and erect with branching, white to cream, (4) colonies were floccose, white and turned into powdery with age, and (5) conidia: hyaline to white, cylindrical, large (T011: 4.7–6.5 × 2.6–3.1 mm, and referent: 3.5–8.0 × 1.5–3.5 µm).

To confirm the authenticity of T011 as *Isaria cicadae*, the construction of *ITS, nrLSU, nrSSU, Rpb1,* and *Tef1*-based phylogeny was performed. According to Mitchell et al. (1995), they suggested that molecular phylogenetic approaches to fungal evolution have proved valuable information toward the goals of understanding the relationship among the specific fungal groups [8]. Additionally, the use of fungal molecular data, including *ITS, nrLSU, nrSSU, Rpb1,* and *Tef1*, for the identification of fungi ushered in a new era of molecular phylogenetic sequence identification in kingdom Fungi [1,12]. In this study, the combination of *ITS, nrLSU, nrSSU, Rpb1,* and *Tef1* genes were applied to strongly strengthen the identification of T011, which was classified as *I. cicadae*. According to phylogenetic analysis, phylogenetic analysis of *ITS, nrLSU, nrSSU, Rpb1,* and *Tef1* yielded consistent topology in different taxa of Clavicipitaceae. The phylogenetic position of T011 was obtained and accepted at subclade level: Cordyceps. Notably, within this clade, the highly supported monophyletic group with referent *C. cicadae* was obtained with high bootstrap value (Bootstrap >95: NJ: 100; MP: 100; ML: 100) and separated this group from other referent taxon in subclade *Cordyceps*, such as *C. ninchukispora*, *C. pruinosa*, and *C. kyusyuensis*. Additionally, T011 formed the group with referent *C. cicadae*, *Cordyceps* sp., and *Isaria* sp. Among them, *Cordyceps* sp. and *Isaria* sp. were proposed using the ancient Chinese name “chanhua” (*Cordyceps chanhua*) [25]. Therefore, based on the phylogenetic analysis, the T011 was identified as the *Isaria cicadae* (anamorph *of C. cicadae*), which was strongly similar to *Cordyceps chanhua*. Therefore, we have successfully applied the phylogenetic analyses based on the concatenated dataset to strengthen the identification of T011, collected in the local coffee garden in Ea Knop Town – Ea Kar District, as *I. cicadae* (anamorph of *C. cicadae*).

## 5 Conclusion

We have successfully applied the phylogenetic analysis of multiple genes of *ITS, nrLSU, nrSSU, Rpb1,* and *Tef1* to demimit sample T011, which was collected in Ea Knop Town – Ea Kar District, Đak Lak Province, was strongly supported as *Isaria cicadae* (anamorph of *C. cicadae*), which was similar to our preliminary identification. This is the first molecular record of *Isaria cicadae* in Vietnam.
